# Deuterium Content of the Organic Compounds in Food Has an Impact on Tumor Growth in Mice

**DOI:** 10.3390/cimb45010005

**Published:** 2022-12-21

**Authors:** Gábor Somlyai, Lajos I. Nagy, László G. Puskás, András Papp, Beáta Z. Kovács, István Fórizs, György Czuppon, Ildikó Somlyai

**Affiliations:** 1 HYD LLC for Cancer Research and Drug Development, H-1118 Budapest, Hungary; 2 AVIDIN Ltd., H-6726 Szeged, Hungary; 3 Department of Public Health, Albert Szent-Györgyi Medical School, University of Szeged, H-6725 Szeged, Hungary; 4 Institute for Geological and Geochemical Research, Research Centre for Astronomy and Earth Sciences, H-1112 Budapest, Hungary; 5 Research Centre for Astronomy and Earth Sciences, MTA Centre of Excellence, H-1121 Budapest, Hungary

**Keywords:** deuterium-depleted water (DDW), deuterium-depleted yolk (DDyolk), anticancer drug development, D/H ratio, production of metabolic water, ketogenic diet

## Abstract

Research with deuterium-depleted water (DDW) in the last two decades proved that the deuterium/hydrogen ratio has a key role in cell cycle regulation and cellular metabolism. The present study aimed to investigate the possible effect of deuterium-depleted yolk (DDyolk) alone and in combination with DDW on cancer growth in two in vivo mouse models. To produce DDyolk, the drinking water of laying hens was replaced with DDW (25 ppm) for 6 weeks, resulting in a 60 ppm D level in dried egg yolk that was used as a deuterium-depleted food additive. In one model, 4T1, a cell line with a high metastatic capacity to the lung was inoculated in the mice’s mammary pad. After three weeks of treatment with DDW and/or DDyolk, the tumor volume in the lungs was smaller in all treated groups vs. controls with natural D levels. Tumor growth and survival in mice transplanted with an MCF-7 breast cancer cell line showed that the anticancer effect of DDW was enhanced by food containing the deuterium-depleted yolk. The study confirmed the importance of the D/H ratio in consumed water and in metabolic water produced by the mitochondria while oxidizing nutrient molecules. This is in line with the concept that the initiation of cell growth requires the cells to generate a higher D/H ratio, but DDW, DDyolk, or the naturally low-D lipids in a ketogenic diet, have a significant effect on tumor growth by preventing the cells from raising the D/H ratio to the threshold.

## 1. Introduction

After the discovery of the heavy isotope of hydrogen, deuterium (D), in the early 1930s, its possible role in living organisms was not investigated for 60 years [1]. The presence of D was ignored; nonetheless, its concentration in the human body is about 12–14 mmol/L (equivalent to 150 ppm). Data gathered in the meantime, however, suggested that D and its ratio to hydrogen (H) has a major impact on several cell processes [2,3]. The first research results, published in 1993, confirmed the key role of D in tumor cell growth and cancer development. An increased D/H ratio in the intracellular space was found to be a key factor in initiating cell growth [4]. Later, results showed the complex nature of the effects of D in living organisms, such as a correlation between the drinking water D level and the susceptibility of humans to depression [5]; stimulation of long-term memory in rats by deuterium-depleted water (DDW) [6] or the reversal of Mn-induced life span decrease in *Caenorhabditis elegans* [7]. Regarding the role of D in cell metabolism, research results confirmed that deuterium depletion enhanced the effect of insulin on Glucose Transporter type 4 (GLUT4) translocation in a dose-dependent manner, and potentiated glucose uptake in diabetic rats, resulting in lower serum glucose, fructose amine, and HbA1c concentrations [8].

Further studies confirmed the role of D in cancer development, in prevention [9], and proved the anticancer effect of deuterium depletion [10,11,12,13,14].

In the experiments revealing the role of deuterium in cell growth, cell metabolism, and physiological changes, the D level was manipulated by the application of DDW.

In the meantime, research confirmed that the D content of organic molecules, such as carbohydrates or lipids, showed significant differences, suggesting that the synthesis pathways of these molecules strongly influence D content. In organic compounds of plants, the D/H ratio may also substantially differ from that of the environment. The explanation for that lies in the metabolic processes of various plants. In plants using the so-called C3 or C4 photosynthetic pathways to fix carbon from the atmosphere, D concentration in the glucose molecules will decrease (vs. environmental water) to different extents. In plants using the C3 carbon fixation pathway (e.g., wheat, rice, barley, or spinach), the glucose D concentration is 135–137 ppm, and in plants using the C4 pathway (maize, sugar cane, millet, and sorghum) it is 141–143 ppm [15,16]. In contrast, plants using Crassulacean Acid Metabolism (CAM) photosynthesis may, under certain circumstances, raise the concentration of deuterium in the photosynthesis products. This means that the deuterium concentration in the human body is substantially affected by the plants in our diet. Investigating the D content of animal lipids led to similar conclusions. Studies revealed significantly reduced D content (down to 90 ppm) in fatty acids and that this effect can be site specific [17,18].

It is obvious that, in the case of a normal diet, the water content of the food will represent the D concentration of the local environment. But that is not the only factor influencing the final D concentration of the body, since the organic molecules of the nutrients also contain both hydrogen and deuterium which appear in the metabolic water after oxidation by the mitochondria.

So, the final D concentration of the cells depends, on the one hand, on the D concentration of the fluid intake, including the water content of the food, but, on the other hand, the D concentration of nutrient molecules also has an effect via the D level of the metabolic water produced in the mitochondria in the oxidative utilization of fats, carbohydrates, and proteins.

Recently, a ketogenic diet has been used as complementary cancer therapy with high efficacy [19]. Based on the proven anticancer effect of DDW, we postulate that the beneficial impact of a ketogenic diet in cancer results from its deuterium-depleting effect, since the mitochondria, when oxidizing fats instead of carbohydrates, produce metabolic water with as low as 118 ppm D concentration, due to the above-mentioned dissimilar D content of the various nutrients [20].

The study presented here aimed to investigate the effect of the D concentration of organic nutrient compounds on tumor development. For this purpose, foods with artificially modified depleted D content were produced, and their effect was tested in two in vivo mouse model systems to evaluate the role of altered D/H ratio of organic molecules and their effect on tumor growth.

## 2. Materials and Methods

### 2.1. Production of Deuterium-Depleted Nutrients, Measurements of D Concentration

Mouse and rat studies indicated that replacing normal drinking water with heavy water increased the D content of organic molecules within a short time [21]. Based on that, the production of deuterium-depleted yolk (DDyolk) was attempted by using DDW as drinking water for laying hens, supposing that during egg formation the D content of proteins and lipids will decrease. DDW was produced by HYD LLC for Cancer Research and Drug Development, Budapest, Hungary, from ordinary tap water by fractional distillation using a Good Manufacturing Practice conform technology. The D concentration was verified by a Liquid-Water Isotope Analyser-24d (manufactured by Los Gatos Research Inc., San Jose, CA, USA) with ±1 ppm precision. Seventeen-week-old Tetra SL hens (n = 90) were kept in a lay house exposing them to an increased day length with artificial light for 14 h per day and were fed with a mixture of grains (corn, wheat, soybean). The D concentration of the drinking water was 25 ppm. The eggs were collected from day zero and the D concentration in both the water content and the dry substance of albumen and yolk fraction was determined. The D concentration of dry substances was determined by mass spectrometry (Finnigan delta plus XP (Thermo Fisher Scientific, Waltham, MA, USA), IAEA-CH-7 and NBS-22 laboratory standards, Institute for Geological and Geochemical Research, CSFK, Budapest, Hungary) with ±1 ppm precision and reported in reference to the Vienna standard mean ocean water, VSMOW, distributed by the International Atomic Energy Agency (<150 ppm) [22,23].

The D concentration reached equilibrium 6−7 weeks after the DDW consumption had started. After 7 weeks of DDW treatment of hens, the eggs were collected and used for the experiments. The albumen and yolk were separated, freeze-dried (to 4.6−5.6% residual moisture), and the yolk was used as a food component for mice.

### 2.2. Preparations Made for per os Treatment of the Mice

Two different in vivo studies using mice were performed (see Section 2.4. for details). For the first one, lasting four weeks, dried yolk (deuterium-depleted and control) was dissolved in distilled water to 0.25 g/mL concentration at 50 °C. The mice received *per os* by gavage 400 µL of the solution daily in the first two weeks of the four-week-long experiment. The mice in the groups receiving the yolk-containing food lost vitality, attributable to the avidin content of the yolk causing biotin deficiency. So, in the second two weeks, the yolk solution was treated at 80 °C for 10 min and the adverse effects disappeared.

In the second experiment, the stress possibly caused by the gavage application was avoided by mixing the dried yolk with powdered semi-synthetic food (VRF1, Akronom Kft., Budapest, Hungary) at 7%. The mix was re-tableted and sterilized for feeding the mice.

### 2.3. Cell Lines

A metastasis-specific mouse mammary carcinoma cell line 4T1 with high metastatic capacity to the lung (purchased from ATCC, Manassas, VA, USA) was grown at 37 °C under 5% of CO_2_ and 100% humidity in RPMI-1640 Medium (Gibco BRL, Carlsbad, CA, USA) containing penicillin (50 IU/mL) (Gibco BRL, Carlsbad, CA, USA), streptomycin (50 mg/mL) (Gibco BRL, Carlsbad, CA, USA), and 10% fetal bovine serum (Gibco BRL, Carlsbad, CA, USA).

An MCF-7 human breast cell line (ATCC) was grown at 37 °C under 5% of CO_2_ and 100% humidity in Eagle’s Minimum Essential Medium (EMEM) (Gibco BRL, Carlsbad, CA, USA) containing penicillin (50 IU/mL) (Gibco BRL, Carlsbad, CA, USA), streptomycin (50 mg/mL) (Gibco BRL, Carlsbad, CA, USA), and 10% fetal bovine serum (Gibco BRL, Carlsbad, CA, USA).

Of both cell lines, the third and fourth passages were used for inoculation. Before inoculation, the cells were trypsinized, washed, and resuspended in sterile PBS. A total of 10^5^ (4T1) or 5 × 10^6^ (MCF7) cells were counted and resuspend in appropriate serum-free medium and injected into the mammary pad in 50 µL volume.

### 2.4. Description of the In Vivo Studies

In the first, 4-week study, 8-week-old female and male BALB/cJ mice (Charles River, Innovo Kft., Hungary) were used. The mice (20 ± 4 g weight) were group-housed (5/cage), fed VRF1 commercial diet ad libitum, and housed in an animal facility under a 12 h light/dark cycle at constant temperature (22 °C) and humidity. The mice were randomly divided into six groups (*n* = 10, 5 males and 5 females) (Table 1). Water was provided ad libitum, with 150 ppm deuterium content (natural level) for the control animals, and with 25 ppm deuterium content for the DDW-treated mice. In this study, 4T1 cells (metastasis-specific mouse mammary carcinoma model cell line; [24]) were used.

On day 0, the cells (100,000/animal in 50 µL) were inoculated into the mice’s mammary pad, and DDW treatment was started. The administration of the yolk solution started on day 1.

Since feeding by gavage with the 400 µL solution of the dried yolk was stressful for the mice, an additional control group was set up (treated with 400 µL normal yolk) to compare the data of the other two treated groups receiving DDW and/or yolk. DU283, a compound with documented anticancer effect, was administered (3 mg/kg bw in 150 µL volume, intraperitoneal, once a day) as a positive control [25]. The growth of the primary tumor was followed on the sixth, eighth, and tenth day of treatment. At the end of the experiment, the lungs were prepared for weight measurement after incubation in formalin. The presence and size of the metastatic tumors were inferred by comparing the weight of treated mice’s lungs to the average lung weight of five healthy, untreated mice; based on the correlation between the lung weight and the number of metastases, reported earlier in a paper by Ying et al. [26].

In the second mice study (Table 2), eight-week-old NSG immunodeficient mice (Charles River, Innovo Kft., 2117 Isaszeg, Hungary) were used. The mice (20 ± 4 g weight) were fed a sterile VRF1 commercial diet and sterile water ad libitum and were housed in individual ventilated cages under sterile circumstances in an animal facility under a 12 h light/dark cycle at constant temperature (22 °C) and humidity. The mice were randomized and three groups (*n* = 10, 5 males and 5 females) were created. The control group consumed normal water ad libitum and dried VRF1-based food supplemented with 7% normal dried yolk (see Section 2.2.). One of the treated groups consumed DDW with 25 ppm D concentration and received the same food as the control group. The second treated group consumed DDW with 25 ppm D concentration and VRF1-based food supplemented with 7% deuterium-depleted dried yolk. Five million MCF-7 cells were inoculated at the start, and the tumor volume and survival were followed up for three months. Tumor volume became measurable around day 14 and was measured every 2–3 days. Each time one mouse perished, the average tumor volume in the three groups was calculated using the previous day’s volume data, divided by the number of mice alive.

### 2.5. Ethical Considerations

The study was performed according to the Institutional and National Animal Experimentation and Ethics Guidelines in possession of an ethical clearance (XXIX./128/2013.; Title: Investigation of effects of macromolecules).

All animals were treated moribund and were euthanized at the observation of the first sign of torment. All operative procedures and animal care conformed strictly to the Hungarian Council on Animal Care guidelines. After treatment, the animals were randomly divided into different groups and kept under standard conditions conforming to ARRIVE (Animal Research: Reporting of in Vivo Experiments) guidelines [27] and the Guide for the Care and Use of Laboratory Animals [28].

## 3. Results

### 3.1. Production of Deuterium-Depleted Food

**Figure 1:**
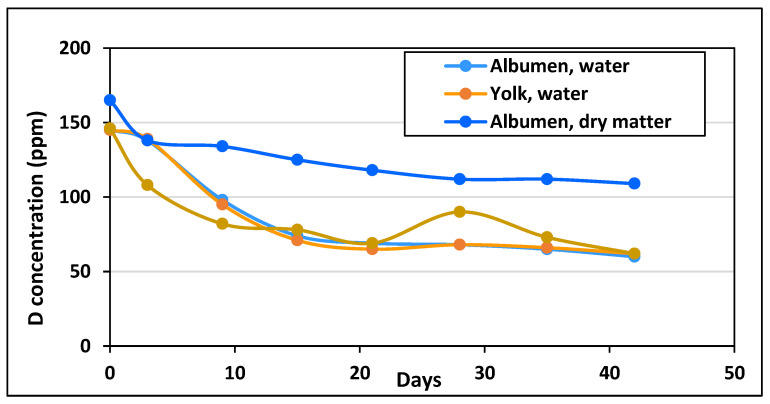
Changes of the D concentration in the water and the organic compounds of eggs after replacement of normal drinking water with 25 ppm DDW (the data represent the average of measurements on two independent samples.).

It was supposed that the replacement of the laying hens’ normal drinking water with DDW of 25 ppm D concentration would influence the D content of the molecules synthesized in the developing eggs. The time course of D levels in water and dry substance of egg white and yolk is shown in Figure 1.

The D concentration of the water extracted from the yolk and egg white was equal, suggesting an equilibrium of D within the liquid phase. There was a sharp 50 ppm decrease in the first 9 days, and a further 30 ppm decrease during the next 12 days, but only a few ppm decrease in the subsequent 3 weeks. The time course of the change of D concentration in egg white and yolk was quite different. The D level of the albumen was higher already before the hens started drinking DDW, and it decreased from 160 ppm to 110 ppm by the forty-second day of the study. The D concentration of the yolk started to decrease rapidly right after the hens started consuming DDW and decreased much faster than the D concentration of the water content of the yolk, reaching equilibrium after only two weeks. By the time of collecting the eggs for the experiments, there was a 50 ppm difference between the D concentration of the egg white (110 ppm) and the yolk (60 ppm).

### 3.2. Tumor Growth in Mice Inoculated with 4T1 Breast Carcinoma Cells

The 4T1 model system was chosen as a cell line with high metastatic capacity to the lung. Therefore, the size of the primary tumor and the weight of the lung metastases were followed.

**Figure 2:**
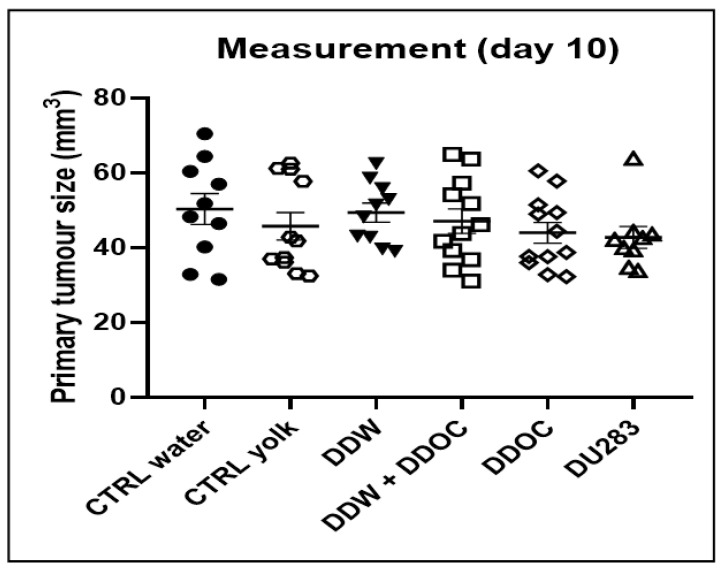
Tumor volume 10 days after inoculation of 4T1 cells (100,000/animal in 50 µL) into the mice’s mammary pad. Dots represent individual data while the horizontal line with error bar shows mean and SD.

In the group consuming DDW and DDyolk, the average primary tumor size was smaller vs. control on days 6, 8, and 10 (9.9 mm^3^, 30.6 mm^3^, 47.0 mm^3^ vs. 15.7 mm^3^, 33.4 mm^3^ 50.3 mm^3^, respectively), but the difference was not significant. Figure 2 shows the data on tumor volumes.

**Figure 3:**
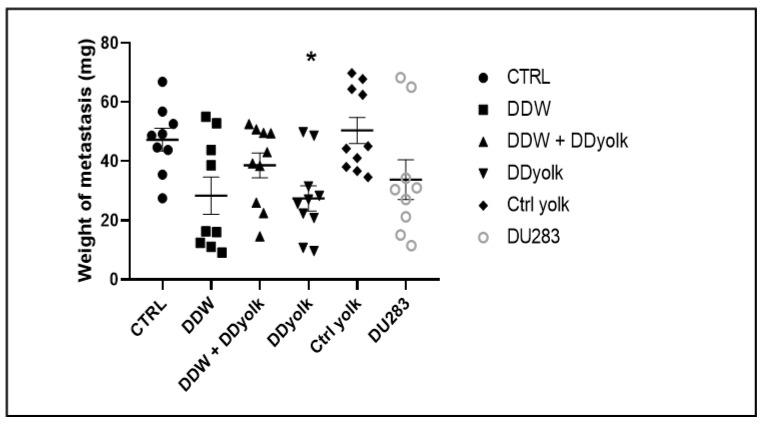
Weight of the metastases in the four treated and two control groups. The average weight of the metastases was lower in all four treated groups but only the group receiving deuterium-depleted yolk (*) showed significant differences (*p* = 0.0354) compared to the group consuming normal water and normal food. Dots represent individual data while the horizontal line with error bar shows mean and SD.

The average weight of the metastases in the lungs and the primary data are shown in Table 3 and Figure 3. In all four treated groups, the tumor weight was smaller compared to the two control groups, but the difference was significant in the group treated with deuterium-depleted yolk (Sidak’s multiple comparisons test, *p* = 0.0354). These differences may be explained with earlier findings showing that DDW inhibits the migration of tumor cells [14].

### 3.3. Tumor Size and Survival in Mice Inoculated with Human MCF-7 Breast Cancer Cell Line

**Figure 4:**
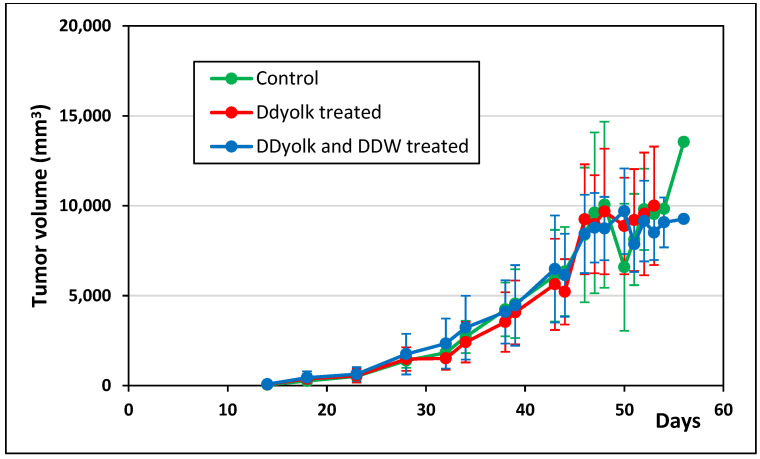
Changes of primary tumor volume in the control and the two treated groups inoculated with the MCF-7 cell line. The primary tumor in the mammary pad was first palpable on day 11.

**Figure 5:**
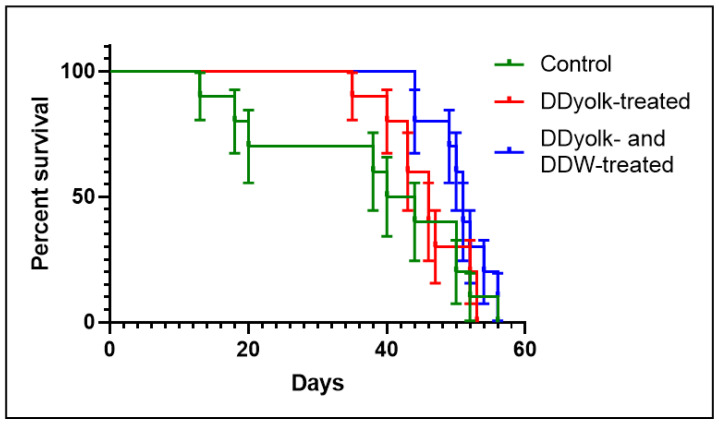
Survival curves of the control and the two treated groups inoculated with the MCF-7 cell line.

The average tumor volume data and survival data are shown in Figure 4 and Figure 5. The tumor volume in the treated groups was somewhat smaller but the difference was not significant. According to the survival data, deuterium depletion delayed the perishment of mice as 30% of them died within 20 days in the control group but within 43 and 49 days in the DDyolk-treated and DDyolk- plus DDW-treated groups, respectively. However, the differences in median survival time were not significant (Logrank test: *p* = 0.0623, Gehan-Breslow-Wilcoxon test: *p* = 0.0689).

These results confirm the anticancer effect of DDW and our assumption that by administration of nutrients as deuterium-depleted organic compounds (DDyolk) to normal food, the anticancer effect of deuterium depletion can be boosted. Increasing the amount of DDyolk in the food may enhance antitumor efficacy.

## 4. Discussion

The consumed food has an undoubtedly significant impact on cell metabolism and physiological processes in living organisms. The effects are typically attributed to the composition of the macronutrients (carbohydrates, lipids, proteins) in foods, whereas the possible role of heavy isotope of hydrogen, deuterium, has not been investigated. To preserve health, the most common dietary approach is to reduce fats and increase carbohydrates within the total caloric intake, arguing that the burden of cardiovascular diseases can be reduced this way.

Our study aimed to investigate the impact of the varying D/H ratio in food on tumor growth. To obtain deuterium-depleted nutrients, the drinking water of laying hens was replaced with DDW and the decrease of D concentration in the eggs was followed. Interestingly, the kinetics of the D level change in yolk and egg white was different which suggested that the distribution of D is strongly determined by biochemical pathways.

The mice studies presented here clearly showed the distinct antitumor effect of DDyolk. The significant weight decreases of lung metastases generated by the 4T1 cancer cells in the first study indicated that deuterium depletion may have inhibited the migration. Inhibition of lung cancer cell migration by DDW in vitro has been described earlier [14]. Tumor growth and survival data of mice inoculated with the MCF-7 breast cancer cell line confirmed our hypothesis that alterations in D concentration in organic compounds of food have an impact on tumor growth. This is related to the role of D in cell growth, namely that well-known molecular metabolic processes lead to an increasing D/H ratio which is responsible for the entering of the cells from the G1 to the S phase [4]. A recent study proved that a higher D/H ratio increased the expression of hundreds of genes with a key role in cell cycle regulation. It was concluded that by keeping the D concentration at a low level using DDW, the expression of these genes, and therefore cell growth, can be kept under control [9].

The application of a ketogenic (very low in carbs and high in fats) diet was tested in cancer patients with convincing clinical evidence of the anticancer effect of this type of nutrition. The changes in the metabolic parameters in patients during such a diet, and the beneficial antitumor effects, are well-documented [29,30,31]. Our results up to now suggest a common link between the antitumor effect of DDW and of ketogenic diet; the deuterium-depleting effect of both.

A carbohydrate-rich diet will result in the production of metabolic water with a D concentration close to the Standard Mean Ocean Water (SMOW) value of 155.75 ppm. However, the more complex the molecules are, such as lipids, the lower their D content, because the biochemical processes in the synthesis of these molecules show a preference to the lighter isotope of hydrogen due to the isotopic effect [2,3,18]. Consequently, increasing the ratio of fats in food intake will result in a lower D concentration of the metabolic water produced by the mitochondria.

The data in Table 4 on the D level in the dry matter of certain common foodstuffs provide evidence of the effect of nutrition on the D concentration within the human body, which in turn affects tumor growth [18,32].

A high-carbohydrate and low-fat diet alone results in a higher average D concentration of the body. Consumed carbohydrates are converted into glucose for immediate energy, and into glycogen and fat as stored energy. Oxidation of glucose, immediately or after storage as glycogen, yields metabolic water with higher D level, whereas lipids become deuterium-depleted during synthesis and yield lower D in metabolic water. The D atoms not included into the lipids will be enriched in other molecules, preferably molecules with hydrogen in the exchangeable position, such as amino- and carboxyl groups of amino acids. This may explain the dissimilar D level in egg yolk and white of the DDW-treated hens (Figure 1).

Malignancies are not the only chronic disease positively influenced by reduced D levels. A recent animal study proved that the optimal D concentration of blood for reducing blood sugar levels in rats was between 125 and 140 ppm, stimulating the translocation of GLUT4 from the cytosol to the cell membrane [8]. The beneficial effect of D depletion was also confirmed in a human phase two clinical trial. DDW (105 ppm D) significantly reduced the fasting glucose level and decreased insulin resistance [33]. Another in vitro study confirmed the impact of the light isotopes of not only hydrogen, but also carbon, oxygen, and nitrogen on enzyme activity [34].

To reduce the incidence of chronic diseases, including malignant tumors, in the human population is a paramount aim. The data presented here prove that the isotopic composition of foods has a major impact on cancer cell growth. These observations raise the need to carry out further preclinical studies and human clinical trials to reveal the mechanism of deuterium depletion and to optimize the D concentration of nutrients to reduce the average D level in the blood from 145–150 ppm to 125–140 ppm.

## Figures and Tables

**Table 1:** Groups and treatments in the first mouse study using the 4T1 cell line.

Group	Type of Treatment		
	Drinking Fluid	Food	Drug
Untreated control	Normal water	VRF1	
Treated	DDW	VRF1	
Treated	DDW	VRF1 + deuterium-depleted yolk	
Treated	Normal water	VRF1 + deuterium-depleted yolk	
Untreated control	Normal water	VRF1 + normal yolk	
Positive control	Normal water	VRF1	DU283

**Table 2:** Groups and treatments in the second mouse study using the MCF-7 cell line.

Groups	Drinking Fluid	Food
Untreated control	Normal water	VRF1 supplemented with normal yolk
Treated	DDW	VRF1 supplemented with normal yolk
Treated	DDW	VRF1 supplemented with deuterium-depleted yolk

**Table 3:** Average weight of the metastases in the formalin fixed lungs in the control and treated groups.

Treatment	Weight of the Metastasis (Milligrams; mean ± SEM, *n* = 10))
Normal water (CTRL)	45.20 ± 4.02
DDW	32.13 ± 6.81
DDW + deuterium-depleted yolk (DDyolk)	37.86 ± 5.47
Normal water + DDyolk *	32.19 ± 4.80 *
Normal water + normal yolk (Ctrl yolk)	46.96 ± 5.30
Normal water + DU283	33.78 ± 6.71

* The tumor weight was significantly decreased compared to CTRL (*p* = 0.0354, Sidak’s multiple comparisons test).

**Table 4:** D concentration of the dry matter of different foods.

Type of Foodstuff	D concentration of Dry Matter (ppm)
Wheat flour	150 ± 1
Table sugar	146 ± 1
Cottage cheese	136 ± 1
Olive oil	130 ± 1
Butter	124 ± 1
Pork fat	118 ± 1

## Data Availability

No data supporting report.

## Abbreviations

D	deuterium
DDW	deuterium-depleted water
DDyolk	deuterium-depleted yolk
SMOW	Standard Mean Ocean Water
